# Canine bufavirus in faeces and plasma of dogs with diarrhoea, China

**DOI:** 10.1080/22221751.2018.1563457

**Published:** 2019-02-11

**Authors:** Jingjiao Li, Li Cui, Xutao Deng, Xiangqian Yu, Zhonghai Zhang, Zhibiao Yang, Eric Delwart, Wen Zhang, Xiuguo Hua

**Affiliations:** a Shanghai Key Laboratory of Veterinary Biotechnology, School of Agriculture and Biology, Shanghai Jiao Tong University, Shanghai, People’s Republic of China; b Department of Microbiology, School of Medicine, Jiangsu University, Zhenjiang, People’s Republic of China; c Blood Systems Research Institute, San Francisco, CA, USA; d Shanghai Pudong New Area Center for Animal Disease Prevention and Control, Shanghai, People’s Republic of China; e Department of Laboratory Medicine, University of California San Francisco, San Francisco, CA, USA

**Keywords:** Viral metagenomics, canine bufavirus, phylogenetic tree, diarrhoea, plasma

**Figure 1.**
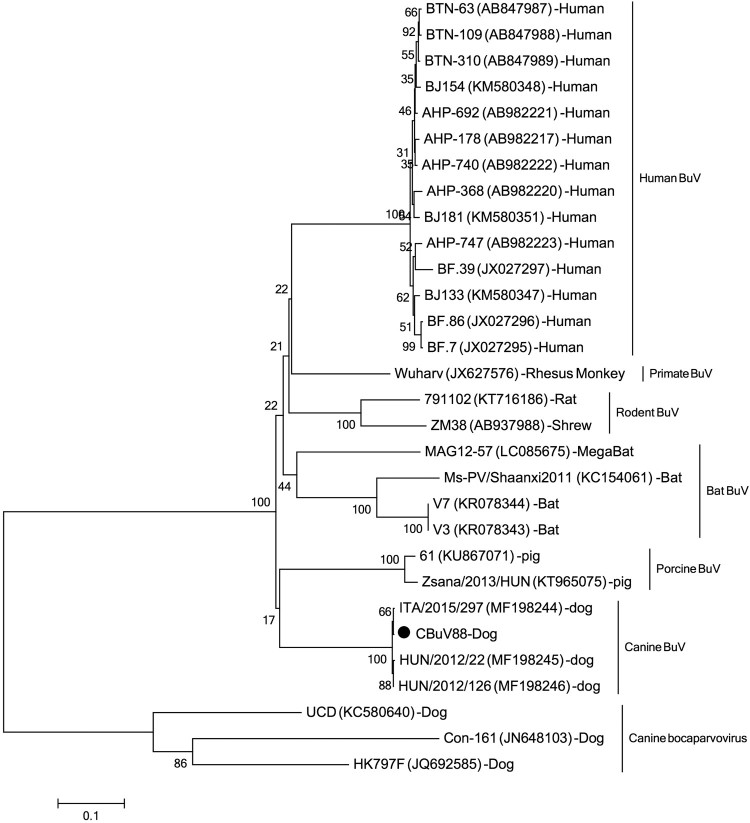
Phylogenetic tree was constructed based on the complete nonstructural protein 1 (NS1) nucleotide sequences using the neighbor-joining method, with bootstrapping over 1,000 replicates, sequences aligning by Clustal W version 2 and phylogenetic analysing by MEAG version 5. Each representative strains were shown in the format: strain name (GenBank number)-species. The strain identified in this study was marked with black dot.

**Dear editor:** Bufavirus (BuV) belongs to the genus Protoparvovirus within the family Parvoviridae [1–4] and was firstly discovered in the stools of diarrhoeic children in 2012 in Burkina Faso [1] which was then classified as members of the primate protoparvovirus species 1 (http://ictvonline.org). Sporadic diarrhoeic cases shedding BuVs have been reported from different countries, suggesting that BuVs might be a possible occasional cause of diarrhoea in humans[1,5,6]. Protoparvoviruses related to BuVs have been detected in humans and other mammals, such as rats, shrews, pigs, dogs, bats and primates [7–12]. Recently, a novel BuV was detected in nasal and oropharyngeal swab of dogs with respiratory signs and faecal samples from diarrhoeic dogs which showed distinct but close genetic relationship to human BuVs [13]. This study showed a statistically significant association of CBuV DNA detection with respiratory signs in dogs from Italy but not with diarrhoea in dogs from Italy and Hungary [13]. MegaBlast search based on the nucleotide sequence of this novel canine BuV against viral metagenomic data we previously generated showed that this virus was also present in a pool of dog faeces consisting of 10 faecal samples collected from pet dogs with diarrhoea in Shanghai, China. This dog faeces pool library had been treated and subjected to library construction as previously reported [14] and sequenced on the Miseq platform generating 1,003,556 unique reads. Twenty-three sequence reads were mapped to the canine BuV genome, which could be assembled into five different contigs. Based on these five contigs, PCR primers was designed to bridge the gaps between contigs to acquire the complete genome, which was named CBuV-88 (GenBank no.: MH645362). Genome was 4319 nt in sequence length including a partial 5′ untranslated region (UTR) (121 nt) and the complete CDSs of NS1 and the nearly complete CDs of VP1, and VP2 genes. Pairwise sequence comparison showed CBuV-88 shared 99% nucleotide sequence identity with canine BuV ITA/2015/297, with only one non-synonymous mutation in the VP2 gene region. Based on the complete genome of CBuV-88 and other related BuV strains, phylogenetic analysis was performed, showing that CBuV-88 closely clustered with canine BuV found in Italy and Hungary (Figure 1).

To investigate the prevalence of CBuV-88 in dogs with diarrhoea in Shanghai, 121 faecal samples from diarrhoeic dogs, 15 faecal samples from healthy dogs, 11 plasma samples from diarrhoeic dogs, and 5 plasma samples from healthy dogs were tested (Table 1). These samples were collected from eight different pet hospitals/kennels, including five different areas of China, Minhang district, Pudong district and Yangpu district of Shanghai, and Yangzhou and Changzhou of Jiangsu. Among the 121 diarrhoeic faecal samples, 105 samples were collected during February 2016 to October 2017, and 16 were collected from diarrhoeic puppies in diarrhoea outbreak in a commercial kennel on June 2018 in Yangpu of Shanghai. The 15 healthy faeces were collected on June 2018 from a different kennel in Pudong of Shanghai. The plasma samples were collected during January 2017 to August 2017 in Pudong of Shanghai (Table 1). Faecal and plasma samples were prepared as our previous report [14,15] and viral DNA were extracted from all the samples using the TaKaRa MinBEST Viral RNA/DNA Extraction Kit (TaKaRa, Japan). A set of semi-nested PCR primers (1FW (5′-GTAAAGAAGAACCAGGACCATC-3′) and 1RW (5′-CACCTGTTCTGAGCAGTTCG-3′) for 1st round PCR and 1RN (5′-CTTCCTGTCCTGTGCCTTG-3′) and 1FW for 2nd round PCR) with an expected product of 609 bp designed based on the VP1 nucleotide sequences of CBuV-88 was used for PCR detection in the canine samples. All the positive BuV samples identified by this initial set of PCR primers was confirmed by amplifying and sequencing another gene region of this virus, using another nested PCR primers (9FW (5′-GGAGACCATGTAAACTACC-3′) and 9RW (5′-CAGCATTAGATAGTTGTTC-3′) for 1st round PCR and 9RN (5′-GGTGACAAATGGGACTCTGG-3′) and 9FN (5′-GTTTCCTTGCCACCACTTGTC-3′) for 2nd round PCR). PCR products were purified by OMEGA Gel Extraction Kit (Omega, USA) and sequenced directly by the Sanger method.

**Table 1.** Information of samples included in this study.

Sampling place	Sample no.		Samples type	Clinical status	Collection date
Minhang, Shanghai	37	Totally 105	Faeces	Diarrhoea	Feb. 2016–Oct. 2017
Pudong, Shanghai	54		Faeces	Diarrhoea	Feb. 2016–Oct. 2017
Changzhou, Jiangsu	8		Faeces	Diarrhoea	Feb. 2016–Oct. 2017
Yangzhou, Jiangsu	6		Faeces	Diarrhoea	Feb. 2016–Oct. 2017
Yangpu, Shanghai	16		Faeces	Diarrhoea	Jun. 2018
Pudong, Shanghai	15		Faeces	Healthy	Jun. 2018
Pudong, Shanghai	11		Plasma	Diarrhoea	Jan. 2017–Aug. 2017
Pudong, Shanghai	5		Plasma	Healthy	Jan. 2017–Aug. 2017

Our results indicated that 51 of the 121 diarrhoeic faecal samples (42.15%) were positive for CBuV-88, which is much higher than that reported for BuVs in humans or other animals [2,5,7]. One of the plasma sample from a diarrhoeic dog was positive, while none of the faecal or plasma samples from healthy dogs was positive. Sequence analysis indicated that these short diagnostic PCR sequences (GenBank number: MH645363- MH645372 for 1p PCR product and MH706930- MH706942 for 9p PCR product) shared >98% nucleotide identities among themselves and to the novel CBuV isolated in Hungary (GenBank accession no. MF198245), indicating that canine BuV strains also circulate in China. Of the 16 faecal samples from the diarrhoea outbreak in the same kennel, only 3 (18.75%) were CBuV positive which is less than the overall positive rate in diarrhoeic dogs in this study. The plasma sample from one male border collie (2.5 month of age) positive for canine BuV suggested that this CBuV can also cause viremia. Although our epidemiologic study was performed based on a limited dog cohort, our data indicated canine BuV might be correlated with diarrhoea in Chinese dogs (*p *≤ 0.01) and further study using larger sample size will be required to elucidate the relationship between canine BuV infection and diarrhoea in dogs.
